# Substitution of Dairy Foods With Plant‐Based Alternatives in German Children and Adolescents: A Modeling Analysis on Nutritional Adequacy and Environmental Sustainability Impact

**DOI:** 10.1002/fsn3.71919

**Published:** 2026-05-23

**Authors:** Eva Hohoff, Karen van de Locht, Ines Perrar, Ute Alexy, Ute Nöthlings

**Affiliations:** ^1^ Institute of Nutritional and Food Sciences ‐ Nutritional Epidemiology University of Bonn Bonn/Dortmund Germany

**Keywords:** adolescents, children, dairy foods, plant‐based‐dairy‐alternatives

## Abstract

The impact of dairy foods substitution with plant‐based dairy alternatives (PBDA) on total daily energy and nutrient adequacy as well as greenhouse gas emission (GHGE) and land use (LU) among children and adolescents was examined. Substitution models replacing dairy foods with PBDA on different levels, stratified by PBDA main ingredients as well as fortified and non‐fortified PBDA and resulting total daily energy and nutrient intakes as well as GHGE (kgCO_2_eq) and LU (m^2^ × year) per day were calculated using data of the dynamic cohort Dortmund Nutritional and Anthropometric Longitudinally Designed (DONALD) study (7676 3 day weighed dietary records from 1072 participants, 3–18 years, 52% boys). Intake of energy and most nutrients decreased significantly with increasing substitution levels. Protein intake exceeded dietary reference intakes (DRI) irrespective of substitution level; vitamin B2 and vitamin B12 fell below the DRI from 50% and 75% substitution level onwards, respectively. The decrease in protein intake was lower for soy‐ than for oat‐based PBDA. Only with 100% substitution of dairy foods with fortified PBDA did median intakes of vitamin B_2_ and vitamin B_12_ reach the DRI. Calcium intake decreased significantly and was well below the DRI at all substitution levels, whereas iron intake increased and almost reached the DRI at a 100% substitution level. GHGE and LU decreased significantly with the increasing degree of substitution. This study shows an improvement of indicators for environmental sustainability when substituting dairy products with PBDA and underlines the potential of fortification of PBDA to improve nutrient adequacy.

## Graphical abstract

Data from the German dynamic cohort DONALD study were used to calculate substitution models replacing dairy foods with plant‐based dairy alternatives (PBDA) on different levels in a pediatric sample. This study shows an improvement of indicators for environmental sustainability when substituting dairy products with PBDA and underlines the potential of fortification of PBDA to improve nutrient adequacy.

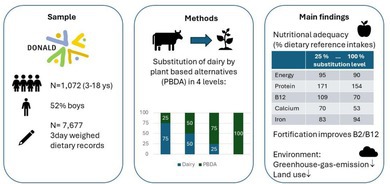

## 1 Introduction

The food system and associated population‐wide dietary intake play a decisive role in the current climate crisis and debate about a more sustainable future. In general, consumption of plant‐based foods causes fewer adverse environmental effects than animal‐source foods (Van de Locht et al. 2024). Furthermore, benefits of plant‐based diets with respect to human health are well established (Willett et al. 2019). However, besides being part of traditional food cultures, dairy foods as one group of animal‐sourced foods provide essential nutrients, for example, protein and calcium, which are particularly important for the vulnerable group of children and adolescents, as requirement of these nutrients is particularly high during growth (Dror and Allen 2014). On the other hand, plant‐based dairy alternatives (PBDA), that is, products based on legumes, grains, or other ingredients that mimic the visual, functional and tasty characteristics of dairy foods, might lower the environmental impact of the diet (Craig et al. 2023; Seves et al. 2017). However, the substitution of dairy foods by PBDA is controversially discussed because of the heterogeneous nutrient profiles of PBDA which clearly deviate from dairy foods (Fructuoso et al. 2021). In fact, PBDA energy and nutrient content varies with respect to the main ingredient, and whether products are fortified or not (Craig and Fresán 2021). Furthermore, PBDA are categorized as processed foods, with in part added sugars, oils or flavors (Scholz‐Ahrens et al. 2020). Up to now, the nutritional impact of the substitution of dairy foods by PBDA has only been investigated in some recent substitution studies (Clegg et al. 2021; Lawrence et al. 2023; Salomé et al. 2021; Seves et al. 2017), two of them including children and adolescents (Clegg et al. 2021; Lawrence et al. 2023). Data on nutrient profiles of PBDA were derived from food composition tables (Lawrence et al. 2023; Salomé et al. 2021; Seves et al. 2017) or a market survey (Clegg et al. 2021). Furthermore most studies did not distinguish between fortified and non‐fortified PBDA (Clegg et al. 2021; Lawrence et al. 2023; Seves et al. 2017) or only considered calcium fortification (Salomé et al. 2021), although nutrient profiles of fortified and non‐fortified PBDA clearly differ (Kersting et al. 2025).

Hence, the current project aimed to tackle this research field by analyzing dairy food intake of children and adolescents quantitatively and qualitatively. Hence, the research objective was to calculate models with varying degrees of substituting consumption of dairy foods with PBDA in the context of total diets. Hereto, substitution models were calculated replacing dairy foods by PBDA in different proportions, based on current intake patterns, that is, food item selection and intake amounts, and stratified by main ingredients as well as fortified and non‐fortified PBDA subgroups. As the global food system causes substantial amounts of anthropogenic greenhouse gas emissions (GHGE) and land use (LU), and dairy foods are among the highest contributors to these environmental sustainability indicators (Van de Locht et al. 2024), both energy and nutrient adequacy as well as GHGE and LU were calculated for the different dietary scenarios.

## 2 Methods

### 2.1 DONALD Study

The DONALD study is an ongoing, dynamic cohort study aimed to investigate nutrition, metabolism, growth and development in healthy infants, children and adolescents aged 3 months to adulthood. Since its start in 1985 in Dortmund, Germany, about 35–40 infants have been enrolled in the study each year. The annual examinations include 3‐day weighed dietary records (3dWR), anthropometric measurements, 24‐h urine samples, lifestyle interviews and medical examinations. In addition, anthropometric data and lifestyle variables as well as socio‐economic status (SES) of the families are collected every four years. Further details are described elsewhere (Perrar et al. 2024).

The ethic committee of the University of Bonn approved the study according to the guidelines of the Declaration of Helsinki (project identification of the most recent version: 185/20). All examinations were carried out with written consent of the participants or their parents. The DONALD study was registered in the German Register of Clinical Trials (DRKS‐ID: DRKS00029092).

### 2.2 Study Sample

**FIGURE 1:**
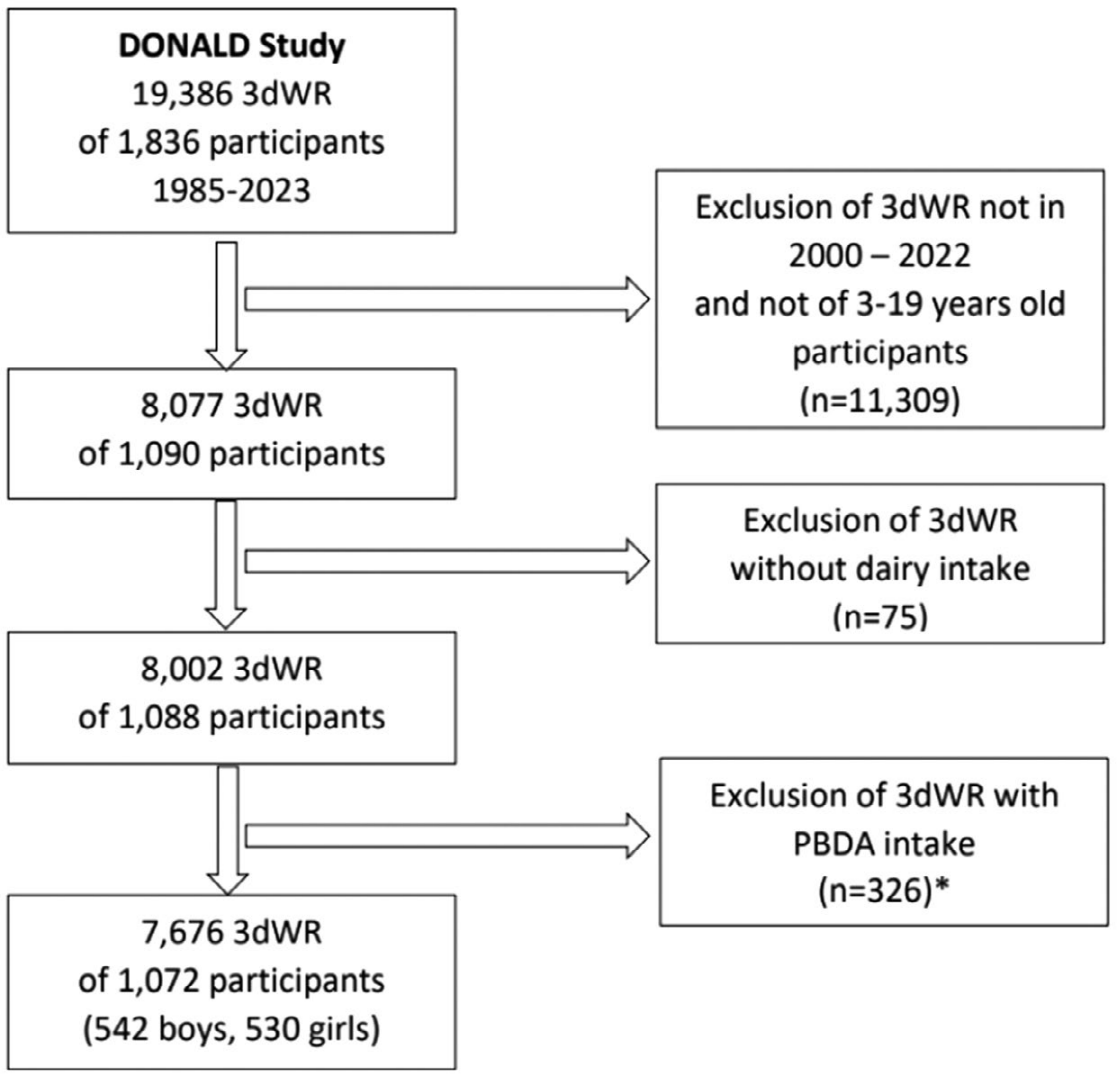
Flow diagram of the selection of dietary records of the DONALD study. 3dWR, three‐day weighing dietary records; PBDA, plant based dairy alternatives. *Data from these records were used to calculate energy and nutrient content of PBDA for simulation models.

The sample of the present investigation is based on all 3dWR of children and adolescents (3 to ≤ 19 years) between 2000 and 2022, as life cycle assessment (LCA) inventory data on GHGE and LU are only valid for more recent years (van de Locht et al., 2024). Those 3dWR without reported dairy foods intake (*n* = 75) were excluded, as it is not possible to calculate a substitution model when dairy intake is zero. Furthermore, for the calculation of the substitution models we excluded those 3dWRs with reported PBDA intake (*n* = 326), as the consumption of PBDA can affect the consumption of dairy. Hence, the final sample consisted of *n* = 7676 3dWR from *n* = 1072 participants (51% boys) (Figure 1).

### 2.3 Dietary Assessment

For the 3dWR, all food and beverages consumed were weighed and recorded on 3 days using electronic food scales. As the participants get older, the recording of data shifts from the parents to the participants themselves: In participants up to 10 years (> 10 years) of age, 65.4% (58.0%) of days were recorded by parents alone. Semi‐quantitative recording (e.g., spoons and cups) was permitted if exact weighing was not possible, for example, when children attended day‐care or school. Information on recipes (ingredients and preparation), types and brands of commercial food products were also required.

Food group, energy and nutrient intakes were calculated using our continually updated in‐house food composition database LEBTAB (Sichert‐Hellert et al. 2007). The composition of basic foods was taken from the German food composition tables BLS 3.02. The energy and nutrient contents of commercial food products, that is, ready‐to‐eat meals, snacks, or PBDA, were estimated by recipe simulation based on the ingredients and nutrients listed including fortification.

For the present evaluation, total energy intake and those nutrients provided in substantial amounts by dairy food products and potential critical in children and adolescents were calculated:

- protein
- fat, saturated (SFA) and polyunsaturated fatty acids (PUFA)
- carbohydrates, total sugars (all mono‐ and disaccharides in foods or beverages), added sugars (all sugars that are added to foods during processing/preparation at home or in manufacture, including sugars from honey, syrups, and fruit juice concentrates (Perrar et al. 2020))
- vitamin B_2_ and vitamin B_12_
- calcium, iron, and iodine

Energy and nutrient intakes were calculated as total daily intake as the individual mean of 3 days.

To calculate ecological sustainability indicators GHGE and LU from total food intake, all food items were linked to the relatively comprehensive SHARP‐indicators database that is commonly used in research (Mertens et al. 2019; Van de Locht et al. 2024). In this database, values of GHGE (kgCO_2_eq per 1 kg of food item) and LU (m^2^ × year per 1 kg of food item) based on life cycle assessment at a European level are included (Mertens et al. 2019). Food items which were not represented in the SHARP‐indicators database were assigned to food items as similar as possible, as published previously (Van de Locht et al. 2024).

### 2.4 Substitution Models

For both dairy foods (e.g., milk, sweetened milk drinks, milk based desserts, yoghurt and other fermented dairy foods, cheese, cream) and PBDA (e.g., plant based milk alternatives, yoghurt alternatives, plant based cheese alternatives, plant based cream), mean energy, nutrients contents and sustainability indicators per 100 g were calculated based on current intake patterns, that is, food item selection and intake amounts. For dairy foods, reported intakes of the main sample were used, and mean energy, nutrients contents and sustainability indicators per 100 g were calculated based on current intake patterns, that is, food item selection and intake amounts. For PBDA, the previously excluded 3dWR (*n* = 326) (Figure 1) with reported PBDA (*n* = 107 different food items) were used. For subgroup analysis, PBDA food items were further grouped by fortified and non‐fortified products and by main ingredient, that is, soy, oats, coconut, rice, almonds and others. PBDA were classified as fortified, when at least one micronutrient was added.

To assess the nutritional implications of the substitution of dairy foods with PBDA, a set of models was calculated in gram‐for‐gram scenarios on a food group basis (e.g., one gram total dairy was replaced by 1 g PBDA, either total PBDA or subgroups), resulting in energy and nutrient intake as well as ecological indicators per day (i.e., individual means of 3 days of recording). Energy and nutrient intake was expressed as the individual achievement of the respective age and sex specific reference values of the German, Austrian and Swiss Nutrition Society (dietary reference intake, DRI) (Deutsche Gesellschaft für Ernährung, Österreichische Gesellschaft für Ernährung, and Schweizerische Gesellschaft für Ernährung 2018) or percentage of energy intake (%E) in case of macronutrients other than protein. In accordance to Farsi et al. (Farsi et al. 2022) each model was calculated for four substitution levels: 25% substitution, 50% substitution, 75% substitution and 100% substitution.

- Model 1: Replacing total dairy foods with the same quantity (in grams) of total PBDA
- Model 2: Replacing total dairy foods with the same quantity (in grams) of fortified PBDA.
- Model 3: Replacing total dairy foods with the same quantity (in grams) of PBDA from different main ingredients (soy, oat), respectively. Due to the small number of PBDAs of different origins consumed, only soy‐ and oat‐based PBDAs were included in the analysis.

Only foods that mainly consist of dairy foods were replaced in the models. Commercial food items with dairy ingredients, for example, frozen pizza with cheese topping, were not considered.

### 2.5 Further Variables

For sample description, participants' body weight status and SES were calculated. Anthropometric measurements (height, weight) were performed by trained nurses according to standard procedures using an electronic scale (Seca 753E; Seca Weighing and Measuring System, ±100 g), and a digital stadiometer (Harpenden, Crymych, UK, ±0.1 cm). The body mass index (BMI [kg/m^2^]) was calculated as the body weight (kg) divided by the square of the body height (m^2^) and sex‐ and age‐specific standard deviation scores (BMI‐SDS) were calculated based on the German reference percentiles for children and adolescents (Kromeyer‐Hauschild et al. 2001). Family SES was assessed using a standardized questionnaire, and a score was calculated (Winkler and Stolzenberg 2009), combining educational and occupational status of the parents (1–7 points, each). In case of different values of mother and father, the highest parental score was used.

### 2.6 Statistical Analyzes

All statistical analyzes were carried out with SAS 9.4.

Sample characteristics were described as median and quartiles (Q1; Q3) for continuous variables. The categorical variables were presented using absolute (*n*) and relative frequencies (%). Differences in the achievement of the DRI for energy and nutrient intake between the current intake and the substitution models were analyzed using analyzes of variance (ANOVA; α = 0.05) and Tukey tests.

## 3 Results

### 3.1 Study Sample

Sample characteristics (1072 individuals, 557 boys, 3–18 years of age, 7676 dietary records) are shown in Table 1. Per participant, a median of six dietary records (min *n* = 1; max *n* = 16) was available for analysis. Median (Q1; Q3) age at time of dietary recording was 10.0 (6.0; 14.0) years. Median (Q1; Q3) BMI‐SDS was 0.00 (−0.61; 0.67), indicating a predominantly normal‐weight sample.

**TABLE 1:** Sample characteristics of *n* = 1072 DONALD participants (age 3 to 18 years) between 2000 and 2022.

**Sample characteristics**		
Participants (% boys)	1072	(52)
Records (% boys)	7676	(50)
n Records per participant	6	(3; 11)
Age (years)	10.0	(6.0; 14.0)
BMI‐SDS^a^	0.00	(−0.61; 0.67)
Socio‐economic status^b^	11.1	(8.4; 11.7)

*Note:* Values are *n* (%) or median (Q1; Q3).

^a^ BMI‐SDS (BMI standard deviation score) calculated based on the German reference percentiles for children and adolescents according to (Kromeyer‐Hauschild et al. 2001).

^b^ Socio‐economic status score according to (Winkler and Stolzenberg 2009), higher score indicates higher SES; 292 missings (values between 1 and 14).

Current median total energy intake (TEI) was slightly below the DRI (median: 96.7%) for low physical activity. Dairy food accounted for around 15.3% of the daily energy intake, but for about a quarter of protein (24.8%), for more than half of calcium (58.3%), and for more than third to vitamin B_2_ (36.7%), vitamin B_12_ (39.2%), and iodine (42.7%). Expressed as %DRI, intake of protein was highest (176%DRI), but DRI for calcium (75.8%DRI), iron (79.9%DRI), and iodine (43%DRI) were not reached (Table 2).

**TABLE 2:** Current dietary intake and ecological indicators of *N* = 7676 3‐day dietary records from *n* = 1072 DONALD participants (age 3 to 18 years) between 2000 and 2022.

Dietary characteristics	Total dietary intake (TDI)				Dairy intake		
	median	(Q1; Q3)			median	(Q1; Q3)	% of TDI
Intake (g/day)	1916	(1522; 2452)			257	(158; 386)	[13.4%]
Energy (kcal/day)	1621	(1323; 1993)	96.7	%DRI^a^	248	(166; 354)	[15.3%]
Protein (g/day)	52.4	(41.6; 65.6)	176.0	%DRI^a^	12.8	(8.3; 18.3)	[24.8%]
Fat (g/day)	60.4	(46.6; 76.6)	34.8	%E^b^	13.2	(8.3; 19.5)	[22.5%]
Saturated Fatty Acids (g/day)	27.3	(20.9; 34.8)	15.7	%E^b^	8.4	(5.3; 12.4)	[30.8%]
Polyunsaturated Fatty Acids (g/day)	7.7	(5.7; 10.5)	4.4	%E^b^	0.4	(0.2; 0.5)	[5.2%]
Carbohydrates (g/day)	209.0	(169.7; 258.0)	53.3	%E^b^	16.7	(9.4; 26.8)	[8.0%]
Total Sugar (g/day)	102.3	(78.7; 132.8)	26.4	%E^b^	15.9	(9.0; 25.2)	[15.5%]
Added Sugar (g/day)	48.1	(32.3; 69.5)	12.3	%E^b^	4.2	(0.0; 9.5)	[8.7%]
Vitamin B_2_ (μg/day)	1304.3	(1015.1; 1717.5)	130.8	%DRI^a^	473.7	(299.4; 712.0)	[36.7%]
Vitamin B_12_ (μg/day)	3.4	(2.5; 4.5)	121.7	%DRI^a^	1.3	(0.8; 1.8)	[39.2%]
Calcium (mg/day)	711.6	(537.9; 940.2)	75.8	%DRI^a^	405.4	(258.6; 587.4)	[58.3%]
Iron (mg/day)	8.7	(6.8; 10.9)	79.9	%DRI^a^	0.3	(0.2; 0.5)	[3.6%]
Iodine (μg/day)	65.4	(48.0; 88.1)	43.0	%DRI^a^	27.1	(15.5; 42.1)	[42.7%]
**Ecological indicators**							
GHGE (kgCO_2_eq)/day	3.74	(2.85; 4.79)			0.8	(0.5; 1.1)	[21.4%]
LU (m^2^/year)/day	4.46	(3.35; 5.87)			0.7	(0.5; 1.1)	[16.6%]

*Note:* Values are median (Q1; Q3). Abbreviations: GHGE = green house gas emission, LU = land use.

^a^ %DRI = Median percent of dietary reference intake per day (Deutsche Gesellschaft für Ernährung, Österreichische Gesellschaft für Ernährung, & Schweizerische Gesellschaft für Ernährung, 2018).

^b^ %E = Median percent of energy intake per day.

### 3.2 Dairy Foods and PBDA Nutrient Content

According to current intake patterns, that is, product selection and consumption amounts, mean energy and nutrient content and ecological indicators per 100 g differed between dairy foods and PBDA (Table 3). Compared to PBDA, dairy foods contained significantly more energy (93 kcal vs. 53 kcal), protein (4.6 g vs. 2.5 g), fat (5.0 g vs. 1.7 g), especially SFA (3.2 g vs. 0.3 g), total sugar (5.8 g vs. 2.9 g), added sugar (1.5 g vs. 0.4 g), calcium (0.14 mg vs. 0.08 mg), iodine (10.6 μg vs. 1.0 μg), vitamin B_2_ (0.18 mg vs. 0.03 mg), and vitamin B_12_ (0.46 μg vs. 0.00 μg). In contrast, PBDA contained more PUFA (0.81 g vs. 0.14 g) and iron (0.64 mg vs. 0.12 mg) than dairy foods.

**TABLE 3:** Mean energy and nutrient content and ecological indicators (per 100 g) of dairy and PBDA according to current consumption pattern from 3‐day dietary records of *n* = 1072 participants (age 3 to 18 years) of the DONALD study between 2000 and 2022.

	Dairy	PBDA	fortified PBDA	non‐fortified PBDA	soy based PBDA	oat based PBDA
*n* products (% fortified)	1308	107 (51)	55	52	51 (67)	23 (48)
**Nutrients**						
Energy kcal/100 g	92.96	52.93	50.12	54.26	56.97	52.93
Protein g/100 g	4.60	2.45	2.96	1.78	3.62	1.71
Fat g/100 g	5.01	1.65	1.52	1.96	1.92	1.29
Saturated Fatty Acids g/100 g	3.18	0.25	0.23	0.27	0.26	0.21
Polyunsaturated Fatty Acids g/100 g	0.14	0.81	0.86	0.81	1.11	0.58
Carbohydrates g/100 g	6.04	6.70	6.72	6.70	3.98	8.14
Total Sugar g/100 g	5.77	2.90	4.00	1.41	3.95	3.67
Added Sugar g/100 g	1.51	0.37	2.54	0.00	3.11	0.00
Vitamin B_2_ mg/100 g	0.18	0.03	0.05	0.02	0.05	0.01
Vitamin B_12_ μg/100 g	0.46	0.00	0.29	0.00	0.12	0.00
Calcium mg/100 g	0.14	0.08	0.12	0.01	0.12	0.01
Iron mg/100 g	0.12	0.64	0.73	0.53	0.86	0.51
Iodine μg/100 g	10.6	1.0	0.81	0.74	0.52	2.52
**Ecological indicators**						
GHGE kgCO_2_eq/100 g	0.28	0.05				
LU m^2^ year/100 g	0.27	0.10				

Abbreviations: GHGE = green house gas emission, LU = land use.

Nutrient contents of PBDA varied between subgroups. Fortified products (*n* = 55, 53%) had higher contents of minerals and vitamins than non‐fortified products (vitamin B_2_ 0.05 vs. 0.02 mg/100 g product, vitamin B_12_: 0.29 vs. 0.0 μg/100 g product, calcium 0.12 vs. 0.01 mg/100 g product, iron 0.73/100 g product vs. 0.53 mg/100 g product, and iodine 0.91 vs. 0.74 μg/100 g product). Soy‐based products (*n* = 51 product, 48%) had higher contents of protein (3.62 g), PUFA (0.26 g), vitamin B_12_ (0.12 μg), calcium (0.12 mg), and added sugar (3.95 g) than oat based products (*n* = 23, 21%): protein 1.71 g, PUFA 0.21 g, vitamin B_12_ 0.00 μg, calcium 0.01 mg, and added sugar (3.67 g) (Table 3).

About two third (67%) of soy based products, and almost half (48%) of oat based products were fortified.

Estimated GHGE and LU per 100 g of dairy foods (0.28 kgCO_2_eq and 0.27 m^2^ × year) exceeded the respective values of PBDA (0.05 kgCO_2_eq and 0.10 m^2^ × year).

### 3.3 Substitution Models

Results of model 1 (replacing total dairy foods with the same quantity of total PBDA) are shown in Table 4. Intake of energy and most nutrients decreased significantly with increasing levels of substitution of dairy foods by PBDA. Exceptions were PUFA, carbohydrates and iron, which showed increasing intakes. Energy intake reached around 90% of DRI in the 100% substitution model. Whereas protein intake exceeded DRI irrespective of replacement level, vitamin B_2_ and vitamin B_12_ fell below the DRI from a replacement level of 50% and 75% onwards, respectively. Calcium intake declined with increasing levels, reaching only 53%DRI with complete substitution of dairy foods. Iodine intake decreased with increasing levels of substitution and only reached 25% of DRI in the 100% substitution. The replacement of dairy foods with PBDA resulted in a significant theoretical reduction in GHGE (up to 19.3% per day) and LU (up to 11.9% per day).

**TABLE 4:** Energy and nutrient intake and sustainability indicators in substitution model 1 replacing 25%, 50%, 75% and 100% of the current total dairy intake with the same amount of PBDA in *n* = 1072 participants (age 3–18 years, *N* = 7676 3‐day dietary records, 2000–2022) of the DONALD study.

	25% PBDA^a^	*p*‐ANOVA^b^	50% PBDA^a^	*p*‐ANOVA^b^	75% PBDA^a^	*p*‐ANOVA^b^	100% PBDA^a^	*p*‐ANOVA^b^
Energy (%DRI)	95.0	**< 0.0001**	93.3	**< 0.0001**	91.6	**< 0.0001**	89.8	**< 0.0001**
Protein (%DRI)	170.6	**< 0.0001**	165.3	**< 0.0001**	159.7	**< 0.0001**	153.9	**< 0.0001**
Fat (%E)	34	**< 0.0001**	33.2	**< 0.0001**	32.3	**< 0.0001**	31.4	**< 0.0001**
Saturated Fatty Acids (%E)	14.8	**< 0.0001**	13.8	**< 0.0001**	12.7	**< 0.0001**	11.6	**< 0.0001**
Polyunsaturated Fatty Acids (%E)	4.8	**< 0.0001**	5.2	**< 0.0001**	5.6	**< 0.0001**	6.0	**< 0.0001**
Carbohydrates (%E)	54.3	**< 0.0001**	55.2	**< 0.0001**	56.3	**< 0.0001**	57.3	**< 0.0001**
Total Sugar (%E)	26.2	0.0680	26.0	**0.0002**	25.7	**< 0.0001**	25.5	**< 0.0001**
Added Sugar (%E)	12.2	0.1817	12.1	**0.0063**	11.9	**< 0.0001**	11.7	**< 0.0001**
Vitamin B_2_ (%DRI)	119.3	**< 0.0001**	108.4	**< 0.0001**	96.9	**< 0.0001**	85.3	**< 0.0001**
Vitamin B_12_ (%DRI)	109.0	**< 0.0001**	96.5	**< 0.0001**	83.1	**< 0.0001**	69.6	**< 0.0001**
Calcium (%DRI)	70.3	**< 0.0001**	64.6	**< 0.0001**	58.9	**< 0.0001**	53.0	**< 0.0001**
Iron (%DRI)	83.3	**< 0.0001**	86.7	**< 0.0001**	90.1	**< 0.0001**	93.6	**< 0.0001**
Iodine (%DRI)	38.8	**< 0.0001**	34.1	**< 0.0001**	29.5	**< 0.0001**	24.5	**< 0.0001**
GHGE (kgCO_2_eq)/day	3.55	**< 0.0001**	3.38	**< 0.0001**	3.19	**< 0.0001**	3.01	**< 0.0001**
LU (m^2^/year)/day	4.30	**0.0228**	4.16	**< 0.0001**	4.04	**< 0.0001**	3.91	**< 0.0001**

*Note:* All models are adjusted for sex and age group. Abbreviations: %DRI = Percent of dietary reference intake per day (Deutsche Gesellschaft für Ernährung, Österreichische Gesellschaft für Ernährung, & Schweizerische Gesellschaft für Ernährung, 2018), %E = Percent of total daily energy intake, GHGE = Green House Gas Emission, LU = Land Use.

^a^ Median.

^b^ *p*‐values refer to the comparison with the current intake; significant *p*‐values are in bold.

With increasing degree of substitution of dairy foods with equivalent amounts of fortified PBDA (models 2), the decrease in the intakes of vitamins and minerals was weaker, but still significant. Even with 100% substitution, median intakes of vitamin B_2_ and vitamin B_12_ reached the DRI. Calcium intake decreased significantly and was well below the DRI on all levels of substitution. The iron intake increased with fortified PBDA and almost reached the DRI at a replacement of 100% (Table 5).

**TABLE 5:** Energy and nutrient intake and sustainability indicators in substitution model 2 replacing 25%, 50%, 75% and 100% of the current dairy intake with the same amount of **fortified PBDA** in *n* = 1072 participants (age 3 to 18 years, *N* = 7676 3‐day dietary records, 2000–2022) of the DONALD study.

	Fortified PBDA (*n* = 55)							
	25% PBDA^a^	*p*‐ANOVA^b^	50% PBDA^a^	*p*‐ANOVA^b^	75% PBDA^a^	*p*‐ANOVA^b^	100% PBDA^a^	*p*‐ANOVA^b^
Energy (%DRI)	94.9	**< 0.0001**	93.1	**< 0.0001**	91.2	**< 0.0001**	89.2	**< 0.0001**
Protein (%DRI)	171.8	**< 0.0001**	167.7	**< 0.0001**	163.2	**< 0.0001**	158.8	**< 0.0001**
Fat (%E)	34.0	**< 0.0001**	33.2	**< 0.0001**	32.2	**< 0.0001**	31.3	**< 0.0001**
Saturated Fatty Acids (%E)	14.9	**< 0.0001**	13.8	**< 0.0001**	12.7	**< 0.0001**	11.6	**< 0.0001**
Polyunsaturated Fatty Acids (%E)	4.8	**< 0.0001**	5.2	**< 0.0001**	5.7	**< 0.0001**	6.1	**< 0.0001**
Carbohydrates (%E)	54.3	**< 0.0001**	55.4	**< 0.0001**	56.5	**< 0.0001**	57.7	**< 0.0001**
Total Sugar (%E)	26.4	0.6170	26.5	0.2959	26.5	0.1016	26.5	**0.0227**
Added Sugar (%E)	12.6	**0.0006**	13.0	**< 0.0001**	13.2	**< 0.0001**	13.5	**< 0.0001**
Vitamin B_2_ (%DRI)	125.8	**< 0.0001**	121.2	**< 0.0001**	116.4	**< 0.0001**	111.8	**< 0.0001**
Vitamin B_12_ (%DRI)	116.8	**< 0.0001**	111.7	**< 0.0001**	106.9	**< 0.0001**	102.2	**< 0.0001**
Calcium (%DRI)	73.5	**< 0.0001**	71.0	**< 0.0001**	68.5	**< 0.0001**	65.7	**< 0.0001**
Iron (%DRI)	83.9	**< 0.0001**	88.0	**< 0.0001**	92.0	**< 0.0001**	96.3	**< 0.0001**
Iodine (%DRI)	38.7	**< 0.0001**	34.0	**< 0.0001**	29.2	**< 0.0001**	24.1	**< 0.0001**

*Note:* All models are adjusted for sex and age group. Abbreviations: %DRI = Percent of dietary reference intake per day (Deutsche Gesellschaft für Ernährung, Österreichische Gesellschaft für Ernährung, & Schweizerische Gesellschaft für Ernährung, 2018), %E = Percent of total daily energy intake.

^a^ Median.

^b^ *p*‐values refer to the comparison with the current intake; significant *p*‐values are in bold.

Results of model 3 are shown in Table 6 (soy‐based PBDA) and Table 7 (oat‐based PBDA). Energy intake decreased significantly with increasing degree of substitution of dairy foods with soy‐ and oat‐based PBDA. The decrease in protein intake with increasing degree of replacement was lower for soy‐based PBDA (100% substitution: 165%DRI) than for oat‐based PBDA (147%DRI). A slightly higher intake of added sugar was observed with soy‐based PBDA (100% substitution: 13.8%E) than oat‐based PBDA (11.4%E).

**TABLE 6:** Energy and nutrient intake and sustainability indicators in substitution model 3 replacing 25%, 50%, 75% and 100% of the current dairy intake with the same amount of **soy‐based PBDA** in *n* = 1072 participants (age 3 to 18 years, *N* = 7676 3‐day dietary records, 2000–2022) of the DONALD study.

	Soy‐based PBDA (*n* = 51)							
	25% PBDA^a^	*p*‐ANOVA^b^	50% PBDA^a^	*p*‐ANOVA^b^	75% PBDA^a^	*p*‐ANOVA^b^	100% PBDA^a^	*p*‐ANOVA^b^
Energy (%DRI)	95.2	**< 0.0001**	93.7	**< 0.0001**	92.1	**< 0.0001**	90.4	**< 0.0001**
Protein (%DRI)	173.4	**0.0001**	170.6	**< 0.0001**	168.1	**< 0.0001**	165.2	**< 0.0001**
Fat (%E)	34.1	**< 0.0001**	33.3	**< 0.0001**	32.4	**< 0.0001**	31.6	**< 0.0001**
Saturated Fatty Acids (%E)	14.8	**< 0.0001**	13.7	**< 0.0001**	12.7	**< 0.0001**	11.5	**< 0.0001**
Polyunsaturated Fatty Acids (%E)	4.9	**< 0.0001**	5.4	**< 0.0001**	6.0	**< 0.0001**	6.5	**< 0.0001**
Carbohydrates (%E)	53.6	**0.0014**	54.0	**< 0.0001**	54.3	**< 0.0001**	54.7	**< 0.0001**
Total Sugar (%E)	26.3	0.7312	26.3	0.5025	26.2	0.3292	26.2	0.2103
Added Sugar (%E)	12.7	**< 0.0001**	13.1	**< 0.0001**	13.4	**< 0.0001**	13.8	**< 0.0001**
Vitamin B_2_ (%DRI)	121.0	**< 0.0001**	111.7	**< 0.0001**	102.0	**< 0.0001**	92.2	**< 0.0001**
Vitamin B_12_ (%DRI)	112.2	**< 0.0001**	102.9	**< 0.0001**	93.3	**< 0.0001**	83.4	**< 0.0001**
Calcium (%DRI)	73.5	**< 0.0001**	71.0	**< 0.0001**	68.5	**< 0.0001**	65.7	**< 0.0001**
Iron (%DRI)	84.8	**< 0.0001**	89.9	**< 0.0001**	94.9	**< 0.0001**	99.5	**< 0.0001**
Iodine (%DRI)	38.6	**< 0.0001**	33.7	**< 0.0001**	28.8	**< 0.0001**	23.5	**< 0.0001**

*Note:* All models are adjusted for sex and age group. Abbreviations: %DRI = Percent of dietary reference intake per day (Deutsche Gesellschaft für Ernährung, Österreichische Gesellschaft für Ernährung, & Schweizerische Gesellschaft für Ernährung, 2018), %E = Percent of total daily energy intake.

^a^ Median.

^b^ *p*‐values refer to the comparison with the current intake; significant *p*‐values are in bold.

**TABLE 7:** Energy and nutrient intake and sustainability indicators in substitution model 3 replacing 25%, 50%, 75% and 100% of the current dairy intake with the same amount of **oat‐based PBDA** in *n* = 1072 participants (age 3 to 18 years, *N* = 7676 3‐day dietary records, 2000–2022) of the DONALD study.

	Oat‐based PBDA (*n* = 23)							
	25% PBDA^a^	*p*‐ANOVA^b^	50% PBDA^a^	*p*‐ANOVA^b^	75% PBDA^a^	*p*‐ANOVA^b^	100% PBDA^a^	*p*‐ANOVA^b^
Energy (%DRI)	95.0	**< 0.0001**	93.3	**< 0.0001**	91.6	**< 0.0001**	89.8	**< 0.0001**
Protein (%DRI)	168.9	**< 0.0001**	161.7	**< 0.0001**	154.4	**< 0.0001**	146.5	**< 0.0001**
Fat (%E)	33.9	**< 0.0001**	32.9	**< 0.0001**	31.8	**< 0.0001**	30.7	**< 0.0001**
Saturated Fatty Acids (%E)	14.8	**< 0.0001**	13.7	**< 0.0001**	12.7	**< 0.0001**	11.5	**< 0.0001**
Polyunsaturated Fatty Acids (%E)	4.7	**< 0.0001**	5.0	**< 0.0001**	5.3	**< 0.0001**	5.6	**< 0.0001**
Carbohydrates (%E)	54.5	**< 0.0001**	55.8	**< 0.0001**	57.1	**< 0.0001**	58.5	**< 0.0001**
Total Sugar (%E)	26.3	0.7162	26.3	0.4739	26.2	0.2925	26.2	0.1717
Added Sugar (%E)	12.1	0.0513	11.9	**< 0.0001**	11.6	**< 0.0001**	11.4	**< 0.0001**
Vitamin B_2_ (%DRI)	118.5	**< 0.0001**	106.8	**< 0.0001**	94.5	**< 0.0001**	81.7	**< 0.0001**
Vitamin B_12_ (%DRI)	109.0	**< 0.0001**	96.5	**< 0.0001**	83.1	**< 0.0001**	69.6	**< 0.0001**
Calcium (%DRI)	65.7	**< 0.0001**	55.5	**< 0.0001**	45.0	**< 0.0001**	33.7	**< 0.0001**
Iron (%DRI)	82.3	**< 0.0001**	84.8	**< 0.0001**	87.4	**< 0.0001**	89.9	**< 0.0001**
Iodine (%DRI)	39.5	**< 0.0001**	35.7	**< 0.0001**	31.7	**< 0.0001**	27.6	**< 0.0001**

*Note:* All models are adjusted for sex and age group. Abbreviations: %DRI = Percent of dietary reference intake per day (Deutsche Gesellschaft für Ernährung, Österreichische Gesellschaft für Ernährung, & Schweizerische Gesellschaft für Ernährung, 2018), %E = Percent of total daily energy intake.

^a^ Median.

^b^ *p*‐values refer to the comparison with the current intake; significant *p*‐values are in bold.

The intake of vitamin B_2_ and B_12_ decreased with increasing levels of substitution, with the soy‐based PBDA showing the smallest difference compared to a dairy foods containing diet. For vitamin B_2_, the DRIs were no longer reached from a 100% substitution, for B_12_ from a 75% substitution. With oat‐based PBDA, the DRI for vitamin B_2_ was already undercut from 75% and for B_12_ from 50% substitution. Calcium intake deteriorated significantly with increasing substitution of dairy foods with PBDA of all types. In the 100% replacement with soy based PBDA calcium intake only reached around 33%DRI. Iron intake increased almost to the level of the DRI in soy‐based and oat‐based variants when dairy foods were completely replaced by PBDA (Tables 6 and 7).

## 4 Discussion

Our study confirms the relevance of dairy food intake for energy and nutrient intake in the current diet of children and adolescents in Germany. In all models, a reduction in energy and nutrient intake (as %DRI) was observed when dairy foods was replaced by PBDA, which became more pronounced with increasing level of substitution. However, in terms of nutrient adequacy of the total diet, this reduction must be viewed in a differentiated way: Irrespective of PBDA subgroup and substitution level, protein intake per day clearly exceeded DRI, so should be no concern based on our data. In contrast vitamin B_2_ and vitamin B_12_ reached DRIs only in the case of partial substitution or with the selection of fortified products. Iron intake improved, in particular in soy‐based models. However, the initially low calcium intake decreased further significantly in all models and was particularly low when dairy foods were substituted with oat‐based PBDA, which were fortified to a lesser extent than soy‐based PBDA. The decrease of the initial low iodine intake was virtually the same in all models, no differences were observed with respect to fortification or main ingredients. Furthermore, our models confirm the expected reduction in GHGE and LU when substituting dairy foods by PBDA.

### 4.1 Nutrient Profile of PBDA

Numerous studies described the nutritional values of PBDA and differences in energy and nutrient content compared to cow's milk (Angelino et al. 2020; Berardy et al. 2022; Clegg et al. 2021; Medici et al. 2023). In a survey across 30 European countries, a total of 309 PBDA were identified, that is, 249 drinks and 60 yoghurts (Medici et al. 2023). As in the present study, the majority were made up of soy (43%) and oat (12%). Calcium was the predominant nutrient used for fortification (49% of products), followed by vitamin B_12_ (39%), vitamin D (36%), and vitamin B_2_ (28%) (Medici et al. 2023). In the present sample, around half of consumed products were fortified, which is in accordance with a European survey (Singh‐Povel et al. 2022). In terms of nutrient content, energy and nutrient of PBDA according to the current consumption pattern of our study population overall confirmed results of other market surveys, for example, from the UK (Clegg et al. 2021), Greece (Katidi et al. 2023), or Italy (Angelino et al. 2020), with lower contents of protein (Katidi et al. 2023; Scholz‐Ahrens et al. 2020), calcium (Clegg et al. 2021; Scholz‐Ahrens et al. 2020) and vitamins, especially vitamin B_2_ and B_12_ (Clegg et al. 2021; Scholz‐Ahrens et al. 2020). Hence, many studies in this context concluded that PBDAs cannot be considered an equivalent substitute for dairy foods (Angelino et al. 2020; Scholz‐Ahrens et al. 2020).

### 4.2 Substitution Models

Nevertheless, the direct comparison of dairy foods and PBDA on a food basis is inconclusive. Inclusion and usage in overall dietary patterns need to be investigated to examine health and environmental aspects of the substitution of dairy foods with PBDA.

In fact, our substitution models have shown that, at least in the case of protein, the lower content in PBDA does not fall below an adequate intake. Protein intake in the DONALD study as well as other studies with children and adolescents in Germany (Alexy et al. 2021; Mensink et al. 2021) is markedly high and clearly exceeded DRIs. Despite a significant reduction in protein intake, the reference values of the total diet were exceeded by 50% in all models, even when replacing dairy foods with the particularly low‐protein PBDA based on oats. However, our models only consider dairy foods for substitution. A simultaneous substitution of other animal source foods with plant‐based alternatives could further reduce protein intake. A recently published model analysis in which meat was replaced by plant‐based alternatives (Farsi et al. 2022), showed that the DRI for protein were no longer achieved with a replacement level of 50% or more, unless tofu, soy and mycoprotein‐based alternatives were used for substitution. Moreover, there are objections that the protein quality of plant‐based foods is lower than that of animal‐based foods. However, a combination of different plant‐based proteins that complement each other in their amino acid profile are usually consumed throughout the day, that is, grains, legumes, nuts and seeds (Herreman et al. 2020; Mariotti 2023). Furthermore, soy protein fall into the high‐quality protein range (Herreman et al. 2020; Hertzler et al. 2020). However, in addition to the amino acid composition, digestibility must also be taken into account when assessing protein quality, which is in general higher in animal protein (mean true nitrogen digestibility: 97%) than in plant protein (88%) (Marinangeli et al. 2021). Thus, an increase in plant protein intake at the expense of animal protein was associated with a decrease in the Protein Digestibility Corrected Amino Acid Score (PDCAAS) in the 2015 Canadian Community Health Survey. In this study, cereal based foods represented the major source of plant protein (Marinangeli et al. 2021). Further studies are needed to determine to what extent these findings can be applied to a diet with a greater diversity of protein sources.

In addition, our models showed a decrease in total energy intake when dairy foods were replaced with PBDA. Hence, to keep total energy intake constant, a higher total food intake would be necessary, which in turn could help to cover the requirement for essential amino acids from other sources than dairy foods (Hertzler et al. 2020).

Along with the decrease in protein intake, there was a decrease in fat intake in our substitution models. Overall fat quality improved due to a reduction in SFA and concomitant increase in PUFA, which has long been recommended for the prevention of cardiovascular diseases. As the development of arteriosclerosis may begin in childhood, the prevention of cardiovascular disease should start early in life (Capra et al. 2023). However, recent findings suggest that the lipid responses following the intake of SFA from dairy foods differ compared to other food sources (Dunne et al. 2024) and evidence for the role of dairy foods intake in the development of cardiovascular disease is inconclusive (Dunne et al. 2024; Givens 2023). Hence, the potential health effects of replacing dairy foods with PBDA remained unclear.

Our analyzes showed an increase in carbohydrate intake in all substitution models. Changes in total and added sugars varied between models with a slight decrease by the substitution with total PBDA, but an increase with fortified and soy‐based PBDA. In accordance with previous analyzes of the DONALD study (Perrar et al. 2020) and other studies (Ivaturi et al. 2024), current sugar intake exceeded the 10%E limit for free sugar in children and adolescents, a substantial proportion originates from sweetened dairy foods (Perrar et al. 2019). The observed reduction through substitution of dairy foods with PBDA is therefore desirable, but this reduction is not very pronounced and depends on the choice of products.

Concerns of insufficient vitamin B_12_ intake with an increased proportion of plant‐based foods in dietary habits have been raised before (Obeid et al. 2019). In our models, intakes of vitamin B_2_ and B_12_ decreased with increasing substitution levels and fell below the DRI when more than half of the dairy foods were replaced by PBDA. The exception were models with fortified PBDA. This underlines the relevance of fortification of PBDA to reach the nutrient content of dairy foods (Clegg et al. 2021), in particular, when meat is also replaced by plant‐based alternatives (Farsi et al. 2022). A study with optimization models of diets free from dairy foods showed that significant changes in the usual dietary habits are required, in particular when dairy food is replaced by non‐fortified PBDA (Taeger and Thiele 2023) to achieve the vitamin B_12_ for vegetarians and also calcium intake for all diets.

Our analyzes confirmed, that dairy food was the main contributor of calcium intake (Bueno and Czepielewski 2008). As the calcium content in non‐fortified PBDA was substantially lower than in dairy foods, the observed decrease in calcium intake with increasing substitution levels could be expected. However, a reduction of calcium was also observed when dairy food was replaced by fortified PBDA. Regularly, the calcium content of fortified PBDA is 120 mg calcium/100 g (Kersting et al. 2025). This value corresponds to the calcium content in cow's milk or yoghurt. In contrast, the dairy food group also contained cheese, which has a higher natural calcium content. Nevertheless, even in the current diet, including dairy foods, calcium DRI were not reached in our sample as well as in representative studies from Germany (Mensink et al. 2021) and international (Shlisky et al. 2022). Furthermore, the bioavailability of added calcium has to be considered, which depends on the chemical form and the presence of absorption inhibitors in the PBDA. The absorption rate of calcium from plant‐based milk alternatives enriched with calcium carbonate from soy is comparable to that of cow's milk; the absorption rate of tricalcium phosphate is lower (Richter et al. 2024).

Iron adequacy improved in our models, with soy‐based PBDA even almost reaching the DRI level at 100% substitution. In adults, iron intake increased when both dairy foods and meat were substituted by plant alternatives, even though most of the iron intake came from a less bioavailable source (Temme et al. 2013). For heme iron, such as that found in meat or fish, bioavailability is estimated to be approximately 15% to 35%, while the absorption rate for non‐heme iron from plant foods ranges from 0.7% to 23% (Deutsche Gesellschaft für Ernährung et al. 2018).

In Germany, dairy foods are an important source of iodine intake, although the iodine content varies depending on the animal feed (Johner et al. 2012). As PBDAs consumed in the DONALD sample were not fortified with iodine, all models showed a significant decline in iodine intake. In fact, a comparison of urinary iodine excretion in UK adults and children ≥ 4 years classified consumers of PBDA as iodine deficient, whereas cows' milk consumers were iodine sufficient (Dineva et al. 2021). A recent modeling study from the UK recommended a fortification of PBDA of ≥ 22.5 and < 45 μg iodine/100 mL would be required to achieve a sufficient iodine intake in particular for children (Nicol et al. 2024). In Germany, however, the DRIs are higher than in other European countries (Deutsche Gesellschaft für Ernährung et al. 2018; EFSA 2014), so that a higher fortification would be necessary.

### 4.3 Sustainability Indicators

Dairy food is one of the main contributors to GHGE and LU in German children and adolescents (Van de Locht et al. 2024). Globally, the dairy food sector alone causes 4.0% of GHGE (Singaravadivelan et al. 2023). In our models, the substitution of dairy foods with PBDA showed a significant theoretical reduction in GHGE and LU. This is in line with the results of a review by Ramsing et al. (2023), in which a generally lower impact of PBDA on environment was stated. However, children and adolescents are an age group with other nutritional needs than adults and a reduction of animal source food with high nutrient densities for environmental sustainability purposes has to ensure energy and nutrient adequacy in this vulnerable groups. However, a recent analysis of DONALD study data indicated that a higher nutrient adequacy was not associated with reduced GHGE and LU (Van de Locht et al. 2024). Hence, a decline in sustainability indicators through a reduction in dairy food consumption must ensure an adequate supply of nutrients. The present study aims to contribute to this issue by identifying potential critical nutrients and the potential of fortification to compensate for shortfalls in these nutrients.

### 4.4 Strength and Limitations

Some strengths and limitations of the present investigation have to be discussed. A major strength is the consideration of the habitual PBDA intake including fortified and non‐fortified products and main ingredients in a pediatric study sample. This approach allows a more realistic calculation of PBDA nutrient profiles than a market survey or the use of food composition tables. However, as food market and thus food consumption patterns change over time, our data only reflect dietary habits of the survey period. Furthermore, we calculated the impact of substitution of dairy foods with PBDA on dairy food key nutrients from the total diet and assessed the adequacy of the resulting diets by comparison with well‐established DRIs. An alternative approach would have been to use the Estimated Average Requirement (EAR) cut‐point method. However, the method does not work with nutrients such as energy where it is known that intakes and requirements are highly correlated (National Academies Press (US) 2000). A further strength is the calculation of two environmental sustainability indicators from the SHARP‐indicators database. However, although the base ingredients used in PBDA have a wide range of environmental impacts, it was not possible to calculate these in detail due to the small quantity of data currently available for determining the environmental sustainability indicators, as also shown in other studies (Berardy et al. 2022). Furthermore, other environmental indicators, for example, water use, might lead to different results but are not included in the SHARP‐indicators database.

The main limitation of our models is the focus on dairy foods, whereas other animal‐based foods were not replaced (Seves et al. 2017; Temme et al. 2013). It was further assumed that intake of other food groups was unchanged. Since the energy intake in the substitution models was below the DRI, it can be assumed that this decrease might be compensated by an increased intake of other food groups and thus might mitigate the observed decrease in vitamin and mineral intakes. On the other hand, the calculation of substitution models that would have kept energy intake constant instead of a gram‐for‐gram scenario would have required larger amounts of PBDA, as the energy content per 100 g of PBDA was lower than that of dairy (Table 3) and thus would have biased the results. In addition, our models did not take into account the variability of nutrient content of dairy food subgroups, for example, high‐ or low‐fat products as well as high or low sugar products. Another limitation is the bioavailability of nutrients, which was not considered in this study. Hence, studies on the long‐term health consequences of a habitual consumption of PBDA are desirable. Furthermore, the present analysis did not consider the use of iodised salt, which is a main source of iodine intake besides dairy foods. In addition, model 2 does not represent an ideal fortification scenario, as it includes any PBDA products with at least one added micronutrient. A further limitation of the present study is the overrepresentation of families with a rather high socio‐economic status in the DONALD study, which limits the generalizability of our results (Perrar et al. 2024).

## 5 Conclusions

Our study has shown that if dairy products are replaced by PBDA and no further dietary modifications are made, indicators for environmental sustainability improve. However, the nutrient intake of some dairy food key nutrients may be insufficient in the diet of children and adolescents. As intake of dairy foods decreases (Hohoff et al. 2021) and PBDA consumption becomes more popular nowadays, families need professional advice how to select the best available products or how to compensate for decrease in key‐nutrient intakes. In particular, since most food based dietary guidelines (FBDG) do not consider PBDA up to now. For example, the recently revised German FBDG recommend for adults an omnivorous diet including two servings of dairy foods per day (Schäfer et al. 2024). In the FBDG of the US, soy drinks fortified with calcium, vitamin A and vitamin D are summarized together with dairy foods in the protein food group. Other PBDA are not included, as the overall nutritional content is regarded as not similar to dairy foods and fortified soy drinks (U.S. Department of Agriculture 2020). Our results are in line with the need to fortify PBDA with calcium, but additionally underline the necessity to fortify PBDA with vitamin B_2_, vitamin B_12_ and iodine, as the decrease in adequacy of these nutrients was at least attenuated. Alternatively, consumers of PBDA should increase the intake of foods high in calcium (e.g., low‐oxalate‐high‐calcium vegetables), vitamin B_2_ (e.g., nutritional yeast), vitamin B_12_ (other animal source food), and iodine (e.g., iodized table salt), or take suitable dietary supplements (e.g., vitamin B_12_).

Our results support current position paper statements emphasizing the importance to select fortified PBDA (Richter et al. 2024) or to ensure that these nutrients are supplied from other foods. Further studies are necessary to assess the long‐term health and nutrient adequacy effects of diets including substantial amounts of PBDA, preferably using biomarkers.

## Author Contributions

**Ines Perrar:** formal analysis, writing – review and editing. **Eva Hohoff:** writing – original draft, formal analysis, data curation. **Ute Nöthlings:** conceptualization, funding acquisition, writing – review and editing. **Karen van de Locht:** data curation, writing – review and editing. **Ute Alexy:** conceptualization, writing – review and editing, supervision.

## Funding

The DONALD Study is financially supported by the Ministry of Science and Research of North Rhine‐Westphalia, Germany. This analysis of the DONALD study data was financially supported by a grant from the ALPRO Foundation and the German Research Foundation (grant no: 509534327). Neither the Alpro Foundation nor the German Research Foundation was involved in the design, implementation, analysis, and interpretation of the data.

## Ethics Statement

This study was conducted according to the guidelines laid down in the Declaration of Helsinki and all procedures involving research study participants were approved by the ethics committee of the University of Bonn. All examinations in the DONALD study were performed after parental and later on also participants' (≥ 16 yeras) written informed consent. The DONALD study was retrospectively registered in the German Register of Clinical Trials (DRKS‐ID: DRKS00029092).

## Conflicts of Interest

The authors declare no conflicts of interest.

## Data Availability

The datasets used and analysed during the current study are available from the corresponding author on reasonable request.
